# Potential Biomarkers of the Turnover, Mineralization, and Volume Classification: Results Using NMR Metabolomics in Hemodialysis Patients

**DOI:** 10.1002/jbm4.10372

**Published:** 2020-05-27

**Authors:** Aline L Baptista, Kallyandra Padilha, Pamella A Malagrino, Gabriela Venturini, Ana CM Zeri, Luciene M dos Reis, Janaina S Martins, Vanda Jorgetti, Alexandre C Pereira, Silvia M Titan, Rosa MA Moyses

**Affiliations:** ^1^ Laboratório de Investigação Médica/LIM 16, Nephrology Division Hospital das Clínicas da Faculdade de Medicina da Universidade de São Paulo São Paulo Brazil; ^2^ Laboratório de Genética e Cardiologia Molecular Instituto do Coração (INCOR), Faculdade de Medicina, Universidade de São Paulo São Paulo Brazil; ^3^ Biosciences National Laboratory LNBio Campinas Brazil; ^4^ Endocrine Unit Massachusetts General Hospital Boston MA USA; ^5^ Endocrine Unit, Medicine, Harvard Medical School Boston MA USA; ^6^ Nephrology Division Hospital das Clínicas da Faculdade de Medicina da Universidade de São Paulo São Paulo Brazil

**Keywords:** BONE HISTOMORPHOMETRY, ANALYSIS/QUANTITATION OF BONE, BIOCHEMICAL MARKERS OF BONE TURNOVER, BONE MODELING AND REMODELING, DISEASES AND DISORDERS OF/RELATED TO BONE, NMR SPECTROSCOPY

## Abstract

Bone biopsy is still the gold standard to assess bone turnover (T), mineralization (M), and volume (V) in CKD patients, and serum biomarkers are not able to replace histomorphometry. Recently, metabolomics has emerged as a new technique that could allow for the identification of new biomarkers useful for disease diagnosis or for the understanding of pathophysiologic mechanisms, but it has never been assessed in the chronic kidney disease–mineral and bone disorder (CKD–MBD) scenario. In this study, we investigated the association between serum metabolites and the bone TMV classification in patients with end‐stage renal disease by using serum NMR spectroscopy and bone biopsy of 49 hemodialysis patients from a single center in Brazil. High T was identified in 21 patients and was associated with higher levels of dimethylsulfone, glycine, citrate, and N‐acetylornithine. The receiver‐operating characteristic curve for the combination of PTH and these metabolites provided an area under the receiver‐operating characteristic curve (AUC) of 0.86 (0.76 to 0.97). Abnormal M was identified in 30 patients and was associated with lower ethanol. The AUC for age, diabetes mellitus, and ethanol was 0.83 (0.71 to 0.96). Low V was identified in 17 patients and was associated with lower carnitine. The association of age, phosphate, and carnitine provided an AUC of 0.83 (0.70 to 0.96). Although differences among the curves by adding selected metabolites to traditional models were not statistically significant, the accuracy of the diagnosis according to the TMV classification seemed to be improved. This is the first study to evaluate the TMV classification system in relation to the serum metabolome assessed by NMR spectroscopy, showing that selected metabolites may help in the evaluation of bone phenotypes in CKD–MBD. © 2020 The Authors. *JBMR Plus* published by Wiley Periodicals, Inc. on behalf of American Society for Bone and Mineral Research.

## Introduction

Chronic kidney disease–mineral and bone disorder (CKD–MBD) is one of the main complications of CKD, universally occurring in those with CKD 3 to 5.^1^, ^2^ It impacts not only bone metabolism, causing renal osteodystrophy, but also has systemic effects, with increased risk of vascular calcification,^3^ uremic arteriolopathy,^4^ cardiovascular disease, and mortality.^5^

Renal osteodystrophy is heterogeneous and complex, including several bone manifestations related to different pathophysiological mechanisms and clinical risks. Renal osteodystrophy diagnosis still relies on the performance of a bone biopsy because no serum or urinary biomarker has been shown to have an accurate performance.^6^, ^7^ The foundation KDIGO (Kidney Disease: Improving Global Outcomes)^6^, ^8^ currently recommends the use of the TMV classification (T = bone turnover, M = bone mineralization, V = bone volume) for the diagnosis of the different types of renal osteodystrophy.^9^

Metabolomics is a technique that allows for the simultaneous identification and quantification of metabolites, ie, small molecules typically with a molecular weight <900 Da. Bone metabolome may yield new biomarkers of disease, as well as indicate molecules and paths involved in pathophysiology. So far, metabolomics studies assessing bone disease have been few; it has been restricted to non‐CKD populations with osteoporosis.^10^, ^11^

The objective of our study was to generate preliminary data on the association of serum metabolites with bone disease defined by the TMV classification.

## Sample and Methods

### Study population

This was an ancillary study of 51 participants from a cross‐sectional study evaluating metabolic syndrome and bone histomorphometry in hemodialysis patients from the Botucatu Medical School Hospital, State University of São Paulo (UNESP), Brazil.^12^ Exclusion criteria were age less than 18 years old, recent start of dialysis treatment (last 6 months), active cancer, advanced liver disease, or the use of glucocorticoids, immunosuppressive or antiretroviral drugs in the past 6 months. Dialysis was performed 3 times a week in 4‐hour sessions. All patients provided written informed consent. The study protocol was reviewed and approved by the local ethics committee and by the National Ethics Board (Cappesq 0046/08 and Plataforma Brasil 3445–2010, respectively).

Serum was collected after 12 hours of fasting on a nondialysis day. Aliquots were stored at −80°C. The following biochemical measurements were performed using standardized techniques: serum total calcium (8.4–10.2 mg/dL, Radiometer ABL7000, Radiometer America Inc., CA, USA); alkaline phosphatase (36–126 U/L, heat inactivation, Thermo Fisher Scientific Inc., MA, USA); phosphorus, albumin, glucose, total cholesterol, HDL‐cholesterol, LDL‐cholesterol and triglycerides (Vitros 950, Johnson & Johnson Chemistry Systems, NJ, USA); intact parathyroid hormone (iPTH, 15–68.3 pg/mL, Abbott Prism System, Abbott Laboratories, USA) and 25 hydroxyvitamin D (30–60 ng/mL, Abbott Prism System, Abbott Laboratories, IL, USA).

### Bone biopsy

Bone biopsy was performed on the iliac crest with an electric trephine, which had an internal diameter of 7 mm, under local anesthesia with lidocaine 2% and sedation with intramuscular midazolam. Previously to biopsy, participants received two courses of tetracycline (20 mg/kg/day) for 3 days, separated by 10 days off medication. The biopsy was performed 2 to 5 days after the end of the antibiotic. The undecalcified bone core was processed using a standardized technique.^13^

Histomorphometric analysis was performed using a semiautomated method with Osteomeasure software (Osteometrics, Inc, Atlanta, GA, USA). The results of bone histomorphometry were categorized according to the TMV classification system described by Moe and colleagues.^8^ Normal turnover was considered when the bone formation rate was 0.07 ± 0.03 μm^3^/μm^2^/day for women and 0.13 ± 0.07 μm^3^/μm^2^/day for men. Participants were then classified as having low bone turnover (lower than −1 SD of the mean reference value) or high bone turnover bone (greater than +1 SD of the mean reference value).^14^ Mineralization was classified as normal or abnormal (mineralization lag time over 50 days). Bone volume was classified as low or normal according to the bone trabecular volume cut‐off value of −1 SD of the mean reference value (21.8 ± 7.2% for women and 24.0 ± 6.1% for men).^15^

### Metabolomics protocol

Our protocol was based on previous studies performed by Beckonert and colleagues^16^ and Malagrino and colleagues.^17^ The selected serum samples underwent deproteinization using ultrafiltration devices (Amicon Ultra 0.5; Millipore, Carrigtwohill, County Cork, Ireland) to remove high molecular weight such as proteins and lipids. Before being used, the filter membranes were washed with deionized water (30 min/cycle, 13,680*g* at 4°C), and this process was repeated 4 times, so that the membrane‐bound residual glycerol was removed. After removing the residual water from the membrane, 350 μL of thawed sample was added, followed by 2 hours of refrigerated centrifugation (4°C) at 13,680*g*. After ultrafiltration, the samples were stored in −80°C freezer until the time of NMR analysis. Thus, the serum filtrate (200 μL) was diluted in 280 μL of deionized water, mixed with 60 μL of phosphate buffer (1M pH 7.4), 5mM of TSP (3‐trimethylsilyl propionic acid‐d4 sodium salt, #269913; Sigma‐Aldrich, St. Louis, MO, USA), and 60 μL of deuterium oxide (D_2_O 99% Sigma; Cambridge Isotope Laboratories, Inc, Tewksbury, MA, USA). Samples were added into 5‐mm proton nuclear magnetic resonance (1H‐NMR) tubes for immediate acquisition.

### NMR acquisition

One‐dimensional 1H NMR spectra were acquired using an Inova NMR AS 600 spectrometer (Agilent Technologies, Inc, Santa Clara, CA, USA) equipped with a 5‐mm cryogenic probe, operating at 599.844‐MHz frequency and a constant temperature of 298 K (25°C). A standard presaturation pulse sequence was performed for water suppression with solvent irradiation on the relaxation delay of 1.5 s and mixing time of 100 ms. NMR spectra were acquired using 256 scans with 64 k points and a spectral width of 13.3 ppm, an acquisition time of 4 s, and a total pulse recycle delay of 5.42 s. The FIDs (free induction decay) were multiplied by an exponential function corresponding to a 0.3‐Hz line broadening prior to Fourier transformation. Spectral phase, baseline correction, and metabolites identification and quantification were performed using Chenomx NMR Suite 7.6 (Chenomx, Inc, Edmonton, Alberta, Canada), a commercial spectral‐fitting software containing an NMR spectral reference library of 304 compounds.

### Statistical analysis

Univariable analysis for clinical and biochemical parameters was done using Mann–Whitney and chi‐square tests. NMR metabolomics identified 64 metabolites (Supplementary Table S1) with 0.7% missing values. Missing values were replaced by half of the minimum positive value of the sample distribution. Metabolites were log‐transformed (generalized log transformation) and evaluated first through primary component analysis (PCA), which showed five extreme outliers. These were excluded, leaving 46 participants for the remaining analysis.

We next performed partial least squares‐discriminant analysis (PLS‐DA) and *t* tests according to the TMV classification as defined above. Selected metabolites were then reassessed in univariable and multivariable logistic regression models (using log base 2 metabolites for ease of interpretation). Receiver operating curves (ROCs) were built to evaluate the performance of selected metabolites in the identification of the TMV classification in comparison with models based on clinical and/or laboratorial variables related to the bone phenotype, using C statistics. The reference models were chosen either as the best model related to the phenotype in question, as was the case for PTH and turnover, or based on literature data when no variables were particularly related to the bone diagnosis, as occurred with mineralization and bone volume (Supplementary Table S2). All tests were bicaudal and values of *p* < 0.05 were considered statistically significant, without adjustment for multiple comparisons. PCA, PLS‐DA, and *t* tests for metabolites were done using MetaboloAnalyst (https://www.metaboanalyst.ca/). Descriptive clinical data and logistic regression models were done using SPSS 25.0 (SPSS, Inc, Chicago, IL, USA). Box plots and ROCs were done using R (packages ggplot2, pROC; R Foundation for Statistical Computing, Vienna, Austria; https://www.r‐project.org/).

## Results

Among the 46 participants, median age was 58 years, 54% of participants were male, and 39% were type 2 diabetic (Table 1). Median iPTH was high (866 pg/mL), and median time on dialysis was 52 months. In terms of bone biopsy, high‐turnover bone disease was diagnosed in 21 (46%), abnormal mineralization in 30 (65%), and low bone volume in 17 (37%).

**Table 1:** Descriptive Characteristics of All Participants and According to Bone Biopsy TMV Classification

	All	Turnover			Mineralization			Volume		
	(*n* = 46)	Low (*n* = 25)	High (*n* = 21)	*p*	Abnormal (*n* = 30)	Normal (*n* = 16)	*p*	Low (*n* = 17)	Normal (*n* = 29)	*p*
Age (years)	58 (44–64)	57 (44–62)	61 (49–65)	0.54	56 (44–62)	63 (49–66)	0.25	59 (56–69)	53 (44–62)	0.16
Sex (male)	25 (54%)	15 (60%)	10 (48%)	0.40	16 (53%)	9 (56%)	0.85	9 (53%)	16 (55%)	0.88
Race (white)	29 (83%)	16 (89%)	13 (77%)	0.33	19 (91%)	10 (71%)	0.14	13 (87%)	16 (80%)	0.61
Diabetes	18 (39%)	12 (48%)	6 (29%)	0.18	14 (47%)	4 (25%)	0.86	8 (47%)	10 (35%)	0.40
Time on dialysis (months)	52 (27–108)	40 (24–84)	72 (33–120)	0.41	45 (24–84)	78 (35–120)	0.28	72 (36–84)	36 (24–120)	0.16
BMI (kg/m^2^)	25.7 (22.7–28.8)	25.9 (23.8–28.4)	25 (22.1–28.8)	0.83	26.4 (23.8–29.5)	24.5 (20.8–27.9)	0.43	23.8 (20.6–27.4)	26.9 (24.1–31.0)	0.03
Albumin (g/dL)	3.8 (3.5–4.1)	3.8 (3.5–4.0)	4.0 (3.6–4.1)	0.41	3.8 (3.5–4.1)	3.8 (3.6–4.0)	0.81	3.7 (3.5–4.1)	3.8 (3.6–4.0)	0.86
Calcium (mg/dL)	9.3 (8.4–10.0)	8.7 (8.2–9.5)	9.7 (9.2–10.2)	0.01	9.0 (8.3–9.7)	9.8 (8.9–10.1)	0.14	8.8 (8.3–9.5)	9.7 (8.6–10.2)	0.05
Phosphate (mg/dL)	5.65 (5.10–6.80)	5.4 (4.2–6.4)	6.6 (5.4–7.4)	0.03	5.7 (4.7–6.9)	5.6 (5.1–6.8)	0.78	5.1 (4.2–5.6)	6.1 (5.4–7.7)	0.01
iPTH (pg/mL)	866 (320–1628)	507 (191–1233)	1374 (392–2315)	0.02	866 (316–1479)	860 (383–2275)	0.39	383 (321–1374)	1164 (392–1643)	0.38
AP (U/L)	171 (100–254)	159 (93–196)	185 (131–286)	0.11	175 (104–241)	144 (110–377)	0.95	185 (114–219)	159 (100–254)	0.44
25‐vit D (ng/mL)	29 (20–36)	26 (20–33)	32 (21–36)	0.54	27 (20–35)	30 (20–37)	0.71	28 (21–37)	30 (20–33)	0.66
Glucose (mg/dL)	95 (78–149)	99 (78–149)	89 (76–121)	0.64	91 (77–160)	95 (82–112)	0.92	99 (83–160)	82 (77–120)	0.12
T. cholesterol (mg/dL)	135 (114–164)	136 (111–161)	133 (122–164)	0.47	139 (114–164)	134 (113–168)	0.66	129 (111–169)	146 (119–163)	0.50
HDL (mg/dL)	40 (31–48)	40 (30–47)	40 (35–49)	0.67	41 (34–48)	35 (30–47)	0.30	42 (36–57)	37 (30–46)	0.08
LDL (mg/dL)	59 (39–84)	56 (38–85)	65 (48–83)	0.42	57 (39–83)	64 (43–90)	0.41	57 (39–82)	65 (39–85)	0.39
Triglycerides (mg/dL)	122 (94–206)	126 (97–200)	119 (94–206)	0.95	123 (95–202)	123 (88–208)	0.94	95 (86–186)	137 (105–234)	0.04
Kt/V	1.30 (1.17–1.54)	1.47 (1.16–1.61)	1.26 (1.18–1.46)	0.6	1.31 (1.16–1.61)	1.28 (1.20–1.51)	0.79	1.38 (1.16–1.51)	1.27 (1.18–1.56)	1.00
Calcitriol	28 (61)	16 (64)	12 (57)	0.76	17 (57)	11 (69)	0.53	11 (64)	17 (59)	0.76
Sevelamer	38 (83)	19 (76)	19 (90)	0.26	24 (80)	14 (87)	0.69	14 (82)	24 (83)	0.99
Ca carbonate	7 (15)	4 (16)	3 (14)	0.99	5 (17)	2 (12)	0.99	1 (6)	6 (20)	0.23
Antihypertensive	26 (57)	12 (48)	14 (66)	0.24	16 (53)	11 (69)	0.36	9 (53)	17 (59)	0.77
Statins	26 (57)	16 (64)	10 (47)	0.37	15 (50)	10 (62)	0.54	10 (59)	15 (52)	0.76

Values described as *n*/% or median/P25 to P75. There were 14 missing values for Kt/V, 11 for race, and 2 for LDL and HDL‐cholesterol.

AP = alkaline phosphatase; iPTH = intact parathyroid hormone; T. cholesterol = total cholesterol.

Table 1 also shows clinical and laboratorial characteristics according to the TMV classification. Among the 21 high‐turnover participants, calcium, phosphorus, and iPTH were higher in comparison to those with low bone turnover. There were no differences in clinical and laboratorial characteristics when comparing those to normal and abnormal mineralization. Participants with low trabecular bone volume had lower BMI, lower calcium, lower phosphorus, and triglycerides (and a trend to lower HDL) in comparison to those with normal bone volume. No differences were seen across the TMV spectrum regarding drugs that could affect bone metabolism, antihypertensive medications, or statins. Of note, no patient was in use of paricalcitol, cinacalcet, bisphosphonates, or glucocorticoids.

### Metabolites—turnover

**Figure 1:**
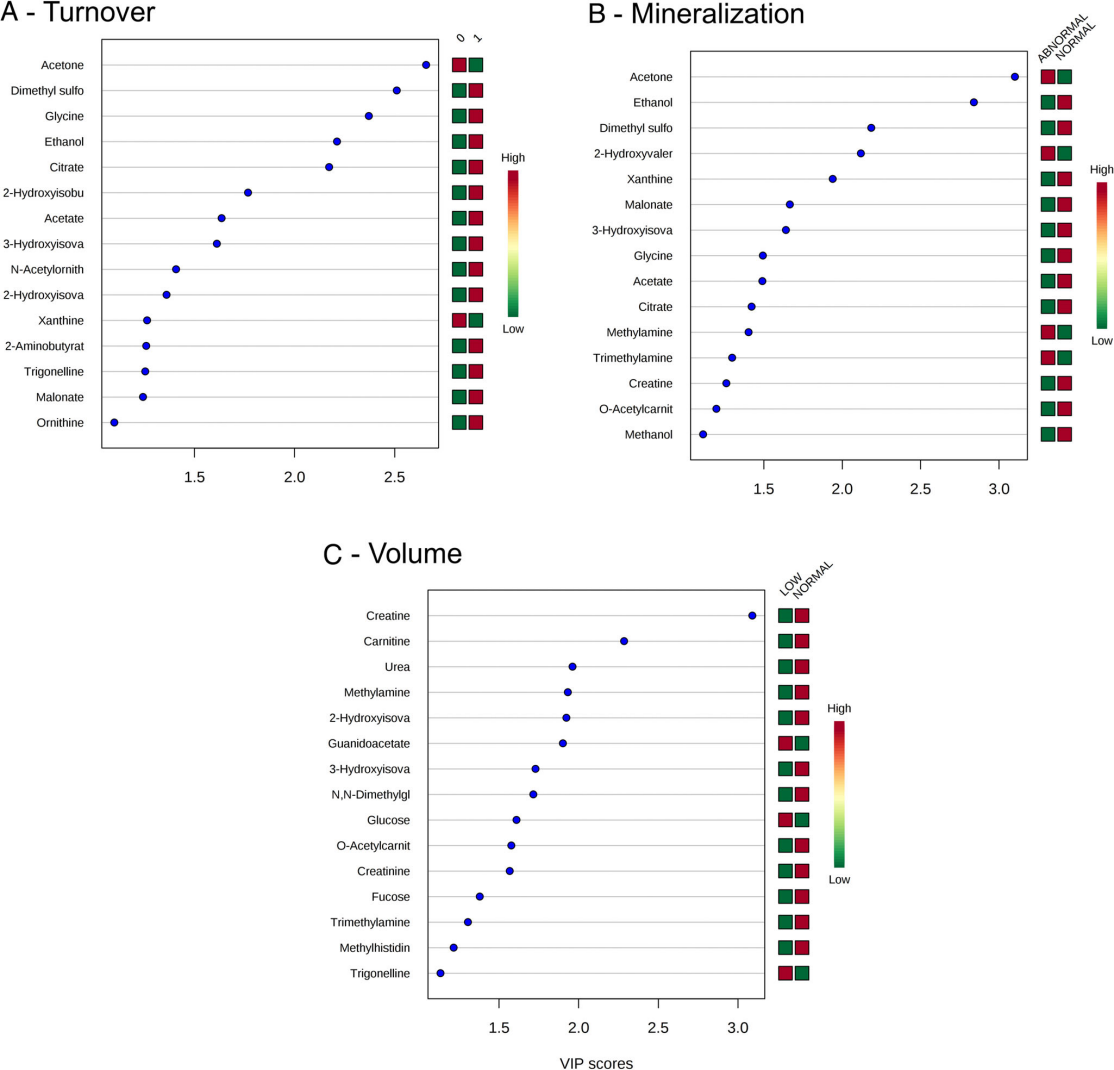
VIP scores of metabolites from partial least squares‐discriminant analysis on the TMV classification. VIP = Variance Importance in Projection; T = turnover; M = mineralization; V = volume.

**Figure 2:**
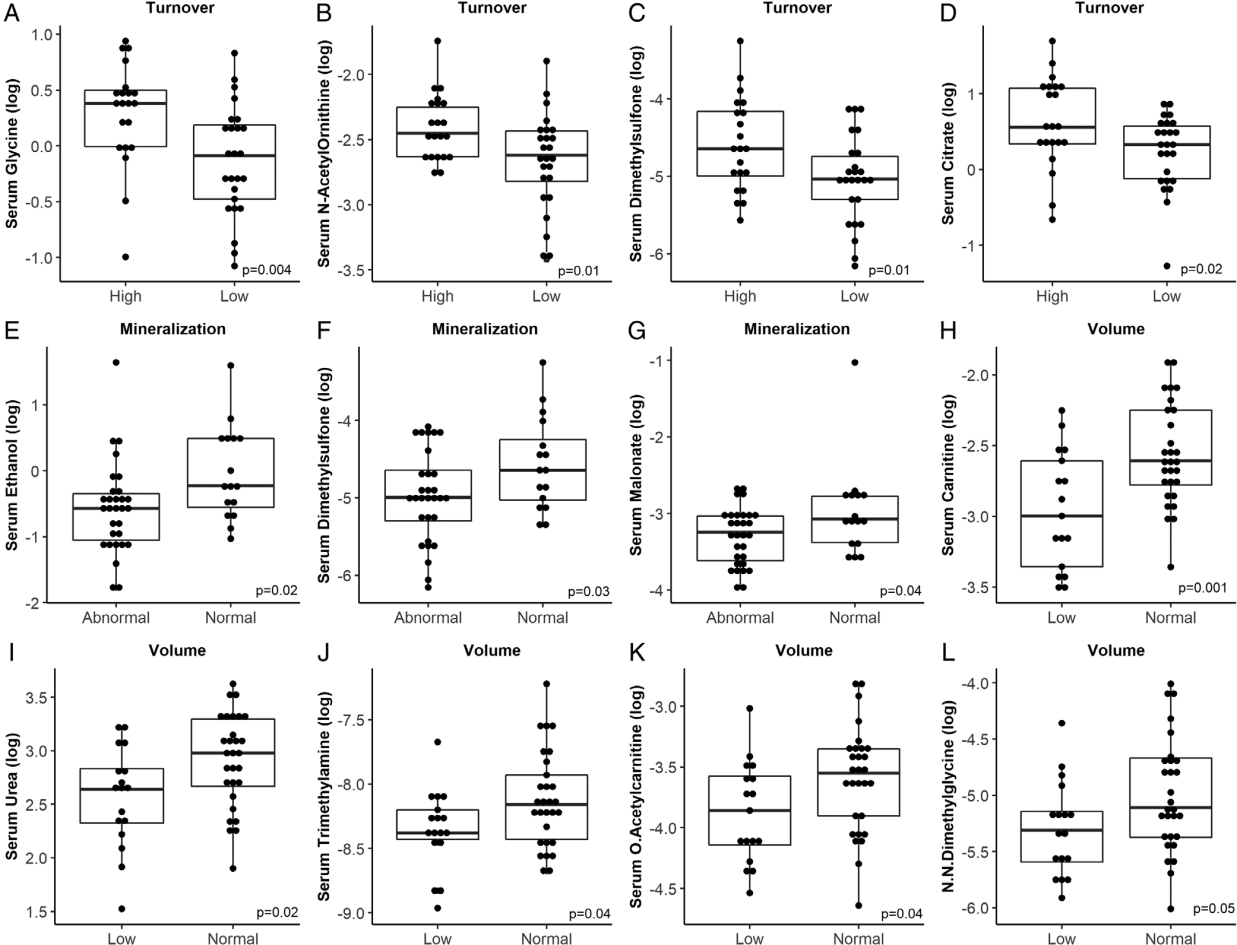
Box plots for selected metabolites related to bone turnover, mineralization, and volume. Box plots for log‐transformed metabolites significantly related to bone turnover (*A*–*D*), bone mineralization (*E*–*G*), and bone volume (*H*–*L*, except for creatinine). Significance was tested with Student's *t* test not adjusted for multiple comparisons.

The PLS‐DA analysis comparing those with high versus those with low bone turnover is shown in Supplementary Fig. S1*A* and Variance Importance in Projection (VIP) scores are shown in Fig. 1 *A*. The top metabolites (VIP above 1.5) associated with bone turnover were acetone, dimethylsulfone, glycine, ethanol, citrate, 2‐hydroxyisobutyrate, acetate, and 3‐hydroxyisovalerate. When the individual metabolites were considered, only glycine (*p* = 0.004), N‐acetylornithine (*p* = 0.01), dimethylsulfone (*p* = 0.01), and citrate (0.02) were different between the two groups, being higher in the high‐turnover participants (Fig. 2 *A–D*).

**Figure 3:**
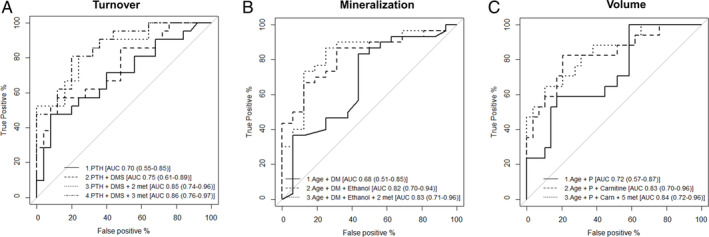
ROCs of logistic regression models based on selected metabolites (with and without traditional biomarkers) on the TMV classification. ROCs were built using estimated predicted values of logistic regression models for bone turnover (*A*, as high turnover versus low turnover), mineralization (*B*, as abnormal versus normal mineralization), and bone volume (*C*, as low versus normal bone volume). For turnover (*A*), the covariates in the models were model 1, PTH; model 2, PTH and dimethylsulfone; model 3, PTH, dimethylsulfone, glycine, and citrate; model 4, same as model 3 plus N‐acetylornithine. C statistics in comparison to model 1 were nonsignificant (*p* = 0.64 for model 2, *p* = 0.12 for model 3, and *p* = 0.10 for model 4). For mineralization (*B*), the covariates were model 1, age, and diabetes; model 2, age, diabetes, and ethanol; model 3, same as model 2 plus dimethylsulfone and malonate. (*C*) Statistics in comparison to model 1 were nonsignificant (*p* = 0.16 for model 2, *p* = 0.21 for model 3). For bone volume (*C*), the covariates in the models were model 1, age, and phosphorus (P); model 2, age, phosphorus, and carnitine; model 3, same as model 2 plus urea, trimethylamine, O‐acetylcarnitine, NN‐dimethylglycine, creatinine. C statistics in comparison to model 1 were nonsignificant (*p* = 0.30 for model 2, *p* = 0.25 for model 3). ROCs = Receiver operating curves; T = turnover; M = mineralization; V = volume.

The univariable logistic regression models confirmed these findings (Table 2 *A*) for the diagnosis of high versus low bone turnover. In model 1, all four metabolites (glycine, N‐acetylornithine, dimethylsulfone, and citrate) were entered in the model and only dimethylsulfone and citrate remained related to bone turnover. This result persisted after adding PTH to the model (model 2; PTH was chosen because it was the only variable related as a reference model). In Fig. 3 *A*, we show the performance of logistic regression models including one to four metabolites for bone turnover phenotype in comparison with a model based solely on PTH. The addition of dimethylsulfone to the PTH model did not increase considerably the AUC, but a moderate increase was observed when dimethylsulfone, glycine, and citrate were added (from 0.70 to 0.85). All these differences, however, were not statistically significant.

**Table 2:** Logistic Regression Models on the TMV Classification of 46 Participants

Metabolites (log2)	Unadjusted			Model 1^a^			Model 2^b^		
	OR	95% CI OR	*p*	OR	95% CI OR	*p*	OR	95% CI OR	*p*
A ‐ TURNOVER									
Glycine	6.94	1.59–30.24	0.01	6.31	0.78–50.70	0.08	6.18	0.77–49.53	0.09
N‐acetylornithine	15.79	1.52–164.40	0.02	5.46	0.18–162.93	0.33	4.78	0.15–156.56	0.38
Dimethylsulfone	3.98	1.24–12.71	0.02	4.88	1.10–21.64	0.04	4.89	1.10–21.84	0.04
Citrate	4.26	1.17–15.60	0.03	6.81	1.16–39.96	0.03	6.24	0.95–41.08	0.06
B ‐ MINERALIZATION									
Ethanol	0.33	0.12–0.89	0.03	0.33	0.11–0.99	0.05	0.23	0.07–0.82	0.02
Dimethylsulfone	0.30	0.10–0.95	0.04	0.39	0.08–1.77	0.22	0.56	0.11–3.03	0.50
Malonate	0.20	0.04–1.11	0.07	0.61	0.09–4.21	0.61	0.49	0.06–4.23	0.52
C ‐ VOLUME									
Carnitine	0.06	0.01–0.43	0.00	0.06	0.00–0.76	0.03	0.07	0.01–0.56	0.01
Urea	0.18	0.04–0.81	0.02	0.37	0.05–2.81	0.34	0.25	0.05–1.17	0.08
Trimethylamine	0.13	0.02–1.00	0.05	0.63	0.04–11.03	0.75	0.14	0.01–1.44	0.10
O‐acetylcarnitine	0.22	0.05–0.99	0.05	1.32	0.10–16.53	0.83	0.21	0.04–1.14	0.07
NN‐dimethylglycine	0.24	0.06–1.03	0.05	0.16	0.02–1.18	0.07	0.25	0.05–1.38	0.11
Creatinine	0.23	0.05–1.03	0.05	2.07	0.17–25.06	0.57	0.19	0.03–1.09	0.06
Creatine	0.52	0.25–1.10	0.09	‐	‐	‐	0.69	0.34–1.40	0.30

Models were built as high versus low for turnover, abnormal versus normal for mineralization, and low versus normal for bone volume.

a Model 1 (adjustment for metabolites): Covariates were glycine, N‐acetylornithine, dimethylsulfone, and citrate for turnover; ethanol, dimethylsulfone, and malonate for mineralization; and carnitine, urea, trimethylamine, O‐acetylornithine, NN‐dimethylglycine, and creatinine for volume.

b Model 2: For turnover, same as model 1 + PTH; for mineralization, same as model 1 + age and phosphorus; for volume, same as model 1 + age and diabetes.

### Metabolites—bone mineralization

The PLS‐DA analysis comparing normal versus abnormal mineralization is shown in Supplementary Fig. S1*B*. The top metabolites were acetone, ethanol, dimethylsulfone, 2‐hydroxyvalerate, xanthine, malonate, 3‐hydroxyisovalerate, glycine, and acetate (Fig. 1 *B*). In the univariable analysis, only ethanol, dimethylsulfone, and malonate were significantly higher in patients with normal mineralization compared to those with abnormal mineralization (Fig. 2 *E–G*). The logistic regression models showed that only ethanol remained significantly associated with mineralization after adjustment for selected metabolites (Table 2 *B*). Ethanol added some performance to the diagnosis of abnormal mineralization in relation to age and diabetes (from 0.68 to 0.82), although this difference was not statistically significant (Fig. 3 *B*).

### Metabolites—bone volume

The PLS‐DA is shown in Supplementary Fig. S1*C*; the VIP scores are listed in Fig. 1 *C*. The top metabolites for discrimination were creatine, carnitine, urea, methylamine, 2‐hydroxyisovalerate, guanidoacetate, 3‐hydroxyisovalerate, NN‐dimethylglycine, glucose, O‐acetylcarnitine, and creatinine. In the univariable analysis, serum concentrations of carnitine, urea, trimethylamine, O‐acetylcarnitine, NN‐dimethylglycine, and creatinine were lower in patients with low bone volume in comparison to those with normal volume (Fig. 2 *H–L*). Again, these results were confirmed in the univariable logistic regression models (Table 2 *C*), but only carnitine remained significantly related to low bone volume (versus normal) after adjustment for other metabolites (model 1) and for phosphorus and age (model 2). The ROC showed that adding carnitine to a model based on age and phosphorus improved the AUC (from 0.72 to 0.83) of low bone volume diagnosis, although this was not statistically significant (Fig. 3 *C*). Adding the other two metabolites (malonate and dimethylsulfone) did not improve the model.

## Discussion

Renal osteodystrophy diagnosis is still dependent on the performance of a bone biopsy, which allows for the measurement of the TMV classification. However, for several reasons, this procedure is not widely performed, therefore limiting our ability to diagnose and follow treatment effects on the bone manifestations of CKD. In this sense, the search for serum biomarkers of disease could be of great benefit. In this study, we explored metabolomics in relation to its association to the TMV classification of bone disease in CKD. Our results point to some metabolites that could potentially have a role in the diagnosis of these conditions.

The PLS‐DAs showed a reasonable ability to discriminate bone turnover, mineralization, and volume, suggesting metabolomic signatures are present in these conditions. When we analyzed individual metabolites, dimethylsulfone, citrate, glycine, and N‐acetylornithine were higher in those with high‐turnover disease. Dimethylsulfone was the only metabolite that showed to be related to turnover independently from the other metabolites. In the ROC, however, dimethylsulfone did not considerably improve performance in relation to a model based solely on PTH. The addition of two metabolites (glycine and citrate) had the best performance, although the difference to the reference model was not statistically significant. It is interesting to note that iPTH alone did not present a good performance for diagnosing bone turnover, a finding that is in accordance with previous studies.^7^, ^18^

In our study, mineralization was associated with lower values of ethanol. No clinical or laboratorial variables were different between participants with and without mineralization defect, and the discriminative performance of these variables for mineralization was very poor. Age and diabetes are known factors to affect bone health^19^ and were chosen as the reference model for predicting mineralization. Adding ethanol to the model seemed to improve the accuracy of a diagnosis of mineralization defect, although this difference did not reach statistical significance.

For bone volume, our results showed association with several metabolites. It is of note that some of the top metabolites identified by PLS‐DA are related to common pathways: the arginine pathway (for guanidoacetic acid, creatine, creatinine, and urea), the methane pathway (trimethylamine and methylamine), in addition to carnitine and O‐acetylcarnitine. Carnitine was the only metabolite that remained significantly associated with bone volume in adjusted models, showing a moderate performance to predict low bone volume when combined with age and phosphorus.

The mechanisms underlying the associations observed could not be addressed in our study. An hypothesis can be made, but needs further exploration and testing. Carnitine is derived from food sources and endogenous metabolism and has a role in fatty acid oxidation.^20^ It is also metabolized by the gut microbiota to trimethylamine. It is associated with sarcopenia and is commonly deficient in hemodialysis patients^21^, ^22^ and other diseases.^23^ In experimental studies, it has been shown to improve bone volume and osteoblast proliferation and differentiation.^24^, ^25^ Ethanol is a biomarker of alcohol consumption and can be produced in small quantities by the intestinal microflora through anaerobic fermentation.^26^ Light‐to‐moderate alcohol consumption has been associated with higher BMD, whereas heavy consumption seems to have the opposite effect.^27^ However, the meaning of decreased ethanol levels in CKD has not been studied, and it is not known whether ethanol is related to changes in the microbiome in CKD. Glycine is a major component of collagen and its association with high‐turnover disease could be a consequence of higher collagen reabsorption. in vitro studies have shown that dimethylsulfone may promote osteogenesis^28^ and osteogenic differentiation in primary bone marrow mesenchymal stem cells.^29^ Citrate is an important metabolite of energy metabolism and also largely present in the bone, where it can be found attached to apatite crystals.^30^, ^31^ A recent study showed that low bone and plasma levels are reduced in osteoporosis.^31^ A study comparing patients with iPTH 150 to 300 pg/mL with patients with iPTH >300 pg/mL showed higher serum values of four intermediate metabolites of the tricarboxylic acid cycle in those with higher iPTH values.^32^ Although all these studies may provide interesting hypothesis on the mechanisms underlying the associations observed, other reasons could be responsible for the associations observed. For example, previous studies have shown that several of these metabolites (glycine, methylsulfone, citrate, and carnitine) are associated with CKD and /or eGFR,^33^, ^34^, ^35^, ^36^ and are therefore related to duration and severity of CKD. In addition, the serum concentration of these metabolites could be related to dialysis efficiency, although in our study KtV was not different between participants.

Our study presents several limitations. First, our sample was small, which limits our statistical power and may lead to overfitting of regression models. In addition, we did not use false discovery rate‐adjusted *p* values, a fact that increases the likelihood of false‐positive results. Second, although Kt/V was not different between TMV groups, we had no data on residual renal function and could not run models with more adjustments. In addition, we had no data on diet intake and could not assess whether metabolite concentration was related to different dietary patterns. Third, we used NMR spectroscopy, a method that brings the advantage of a reliable quantitative measure of metabolites, but is limited in terms of numbers of metabolites being captured in comparison with gas chromatography or liquid chromatography. All these limitations imply that our results are more hypothesis‐generating than conclusive, with need of replication and validation.

In summary, our results suggest some potential metabolites related to turnover, bone volume, and mineralization in CKD patients on hemodialysis. Further studies are needed to validate these findings and to test the accuracy of these biomarkers in a larger population.

## Disclosures

All authors state that they have no conflicts of interest.

## Supporting information


**Appendix S1** **:** Supporting information.
