# Supplementary material for: Potential Biomarkers of the Turnover, Mineralization, and Volume Classification: Results Using NMR Metabolomics in Hemodialysis Patients
Source: JBMR Plus. 2020 May 27;4(7):e10372. doi: 10.1002/jbm4.10372 (PMC7340447; doi:10.1002/jbm4.10372)

Supplementary Material

“Potential biomarkers of the TMV classification: results using NMR-metabolomics in patients under hemodialysis”

Aline L. Baptista, MD[1], Kallyandra Padilha[2], Pamella A. Malagrino[2], Gabriela Venturini[2], Ana C. M. Zeri[3], Luciene M. dos Reis[1], Janaina S. Martins, MD, PhD[4-5], Vanda Jorgetti, MD, PhD[1], Alexandre C. Pereira, MD, PhD[2], Silvia M. Titan, MD, PhD6[6], Rosa M. A. Moyses, MD, PhD[1].

1. Laboratório de Investigação Médica / LIM 16, Nephrology Division, Hospital das Clínicas da Faculdade de Medicina da Universidade de São Paulo, São Paulo, Brazil
2. Laboratório de Genética e Cardiologia Molecular, Instituto do Coração (INCOR), Faculdade de Medicina, Universidade de São Paulo, São Paulo, Brazil
3. Biosciences National Laboratory, LNBio, Campinas, SP, Brazil
4. Endocrine Unit, Massachusetts General Hospital, Boston, USA
5. Harvard Medical School, Boston, Massachusetts, USA
6. Nephrology Division, Hospital das Clínicas da Faculdade de Medicina da Universidade de São Paulo, São Paulo, Brazil

For Review Only

Supplementary Table 1. List of the 64 metabolites identified through NMR-spectroscopy in the 46 participants.

Metabolites (mg/dL)	Median	P25 - P75
alpha2Aminobutyrate	0.05	(0.04 - 0.06)
alpha2Hydroxybutyrate	0.12	(0.11 - 0.17)
alpha2Hydroxyisobutyrate	0.01	(0.01 - 0.02)
alpha2Hydroxyisovalerate	0.03	(0.02 - 0.04)
alpha2Hydroxyvalerate	0.14	(0.10 - 0.16)
alpha3Hydroxybutyrate	0.21	(0.12 - 0.45)
alpha3Hydroxyisovalerate	0.02	(0.01 - 0.02)
alpha5Aminolevulinate	0.14	(0.10 - 0.17)
Acetate	0.13	(0.10 - 0.20)
Acetone	0.01	(0.01 - 0.02)
Alanine	1.32	(1.05 - 1.49)
Asparagine	0.27	(0.23 - 0.31)
Betaine	0.32	(0.25 - 0.40)
Caffeine	0.08	(0.06 - 0.11)
Carnitine	0.16	(0.13 - 0.18)
Choline	0.13	(0.10 - 0.16)
Citrate	1.34	(1.10 - 1.69)
Creatine	0.13	(0.09 - 0.20)
Creatinephosphate	0.28	(0.24 - 0.33)
Creatinine	3.18	(2.29 - 3.74)
Dimethyl sulfone	0.03	(0.03 - 0.05)
Dimethylamine	0.04	(0.04 - 0.05)
Ethanol	0.72	(0.55 - 0.98)
Formate	0.06	(0.05 - 0.07)
Fucose	0.13	(0.11 - 0.16)
Glucose	36.93	(31.22 - 50.73)
Glutamine	3.11	(2.68 - 3.44)
Glycerol	0.67	(0.55 - 0.82)
Glycine	1.12	(0.82 - 1.36)
Glycolate	0.08	(0.07 - 0.10)
Guanidoacetate	0.20	(0.15 - 0.33)
Histidine	0.46	(0.38 - 0.53)
Isobutyrate	0.02	(0.02 - 0.03)
Isoleucine	0.29	(0.24 - 0.37)
Lactate	6.01	(4.65 - 7.73)
Leucine	0.51	(0.41 - 0.60)
Lysine	0.69	(0.58 - 0.82)
Malonate	0.11	(0.09 - 0.12)
Methanol	0.20	(0.17 - 0.25)

For Review Only

Metabolites (mg/dL)	Median	P25 - P75
Methionine	0.13	(0.11 - 0.14)
Methylamine	0.00	(0.00 - 0.01)
NNDimethylglycine	0.03	(0.02 - 0.04)
NAcetylornithine	0.18	(0.16 - 0.20)
NIsovaleroylglycine	0.06	(0.05 - 0.07)
NMethylhydantoin	0.02	(0.01 - 0.02)
OAcetylcarnitine	0.08	(0.06 - 0.09)
Ornithine	0.29	(0.23 - 0.35)
Oxypurinol	0.49	(0.41 - 0.60)
Phenylalanine	0.50	(0.39 - 0.59)
Proline	1.18	(0.99 - 1.48)
Propyleneglycol	0.04	(0.03 - 0.04)
Pyruvate	0.20	(0.14 - 0.26)
Succinate	0.04	(0.04 - 0.05)
Threonate	0.37	(0.26 - 0.48)
Threonine	0.69	(0.58 - 0.83)
Trigonelline	0.10	(0.08 - 0.14)
Trimethylamine	0.00	(0.00 - 0.00)
TrimethylamineNoxide	0.20	(0.13 - 0.26)
Tyrosine	0.34	(0.31 - 0.43)
Urea	7.05	(5.40 - 8.71)
Valine	0.87	(0.70 - 1.03)
Xanthine	0.16	(0.10 - 0.25)
myoInositol	2.12	(1.70 - 2.86)
Tau-Methylhistidine	0.18	(0.15 - 0.21)

For Review Only

Supplementary Table 2. Logistic regression models of clinical and laboratorial variables on the TMV classification of 46 participants.

	B	S.E.	OR	Low 95% CI	High 95% CI	p
A - TURNOVER						
Univariate						
Sex (male)	-0.50	0.60	0.61	0.19	1.96	0.40
Age (years)	0.00	0.02	1.00	0.96	1.05	0.83
Diabetes	-0.84	0.63	0.43	0.13	1.48	0.18
Calcium (mg/dL)	0.63	0.31	1.88	1.03	3.43	0.04
Phosphate (mg/dL)	0.44	0.20	1.56	1.05	2.31	0.03
iPTH (pg/mL)	0.0009	0.0004	1.0009	1.0001	1.0016	0.0222
25-vit. D (ng/mL)	0.01	0.03	1.01	0.96	1.07	0.72
Multivariable (stepwise)*						
iPTH (pg/mL)	0.0013	0.0005	1.0013	1.0004	1.0022	0.0066
B - MINERALIZATION						
Univariate						
Sex (male)	-0.12	0.62	0.89	0.26	3.01	0.85
Age (years)	-0.02	0.02	0.98	0.93	1.03	0.35
Diabetes	0.97	0.68	2.62	0.69	10.02	0.16
Calcium (mg/dL)	-0.27	0.28	0.76	0.44	1.31	0.33
Phosphate (mg/dL)	-0.09	0.16	0.91	0.66	1.25	0.57
iPTH (pg/mL)	-0.0004	0.0003	0.9996	0.9990	1.0003	0.2865
25-vit. D (ng/mL)	-0.01	0.03	0.99	0.94	1.05	0.81
BMI (kg/m^2)	0.01	0.06	1.01	0.90	1.12	0.92
Albumin (g/dL)	0.19	0.69	1.20	0.31	4.63	0.79
C - VOLUME						
Univariate						
Sex (male)	-0.09	0.61	0.91	0.28	3.04	0.88
Age (years)	0.03	0.02	1.03	0.98	1.08	0.24
Diabetes	0.52	0.62	1.69	0.50	5.73	0.40
Calcium (mg/dL)	-0.38	0.28	0.69	0.40	1.18	0.17
Phosphate (mg/dL)	-0.52	0.22	0.59	0.38	0.92	0.02
iPTH (pg/mL)	-0.0003	0.0004	0.9997	0.9990	1.0004	0.4012
25-vit. D (ng/mL)	0.01	0.03	1.01	0.96	1.07	0.64
BMI (kg/m^2)	-0.12	0.07	0.89	0.78	1.01	0.08
Albumin (g/dL)	-0.04	0.68	0.96	0.26	3.61	0.95

Models were built as high versus low for T, abnormal versus normal for mineralization and low versus normal for bone volume. * all variables from univariate models depicted entered in a stepwise regression model.

For Review Only

Supplementary Figure 1. PLS-DA of metabolites according to the TMV classification in 46 participants.

A - Turnover

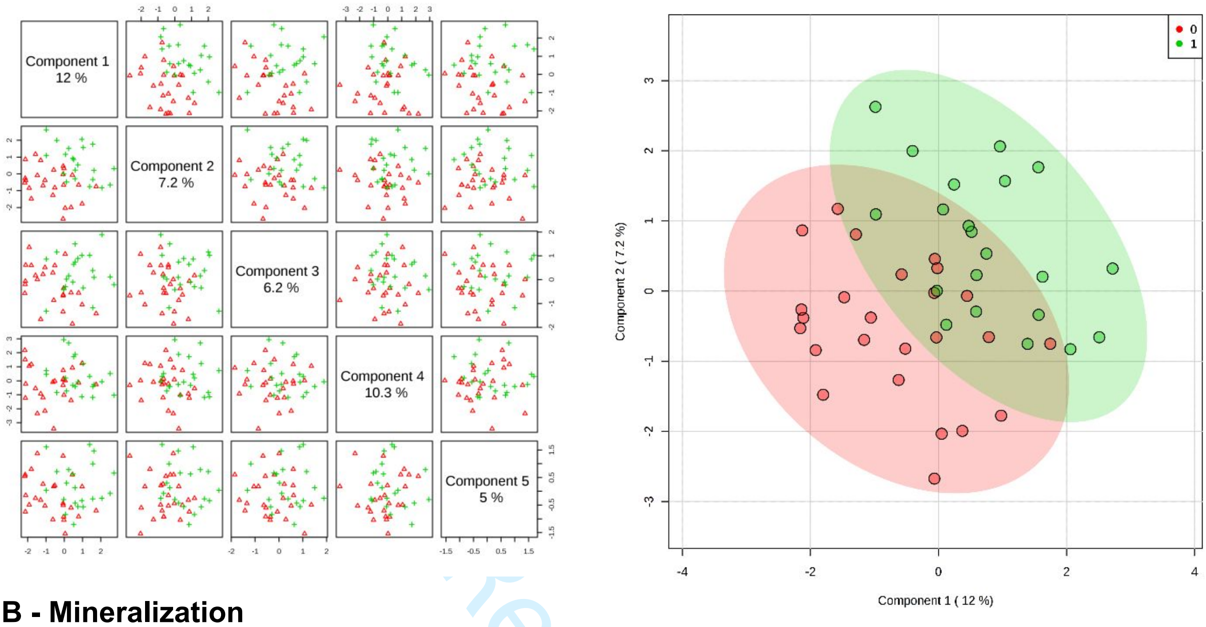

B - Mineralization

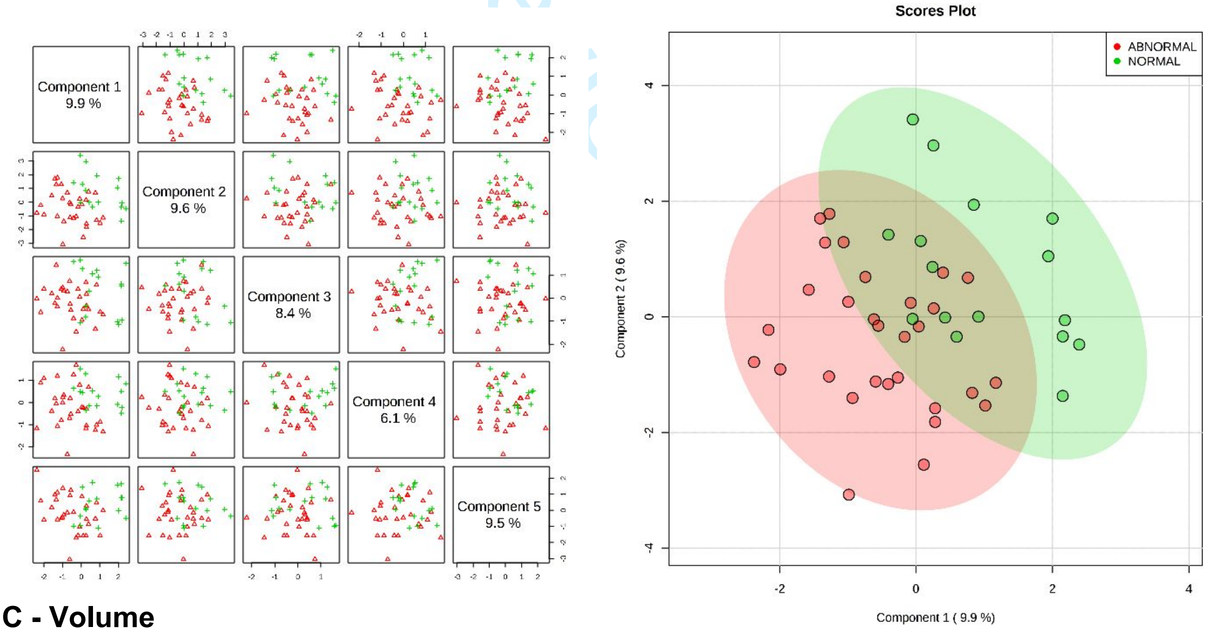

C - Volume

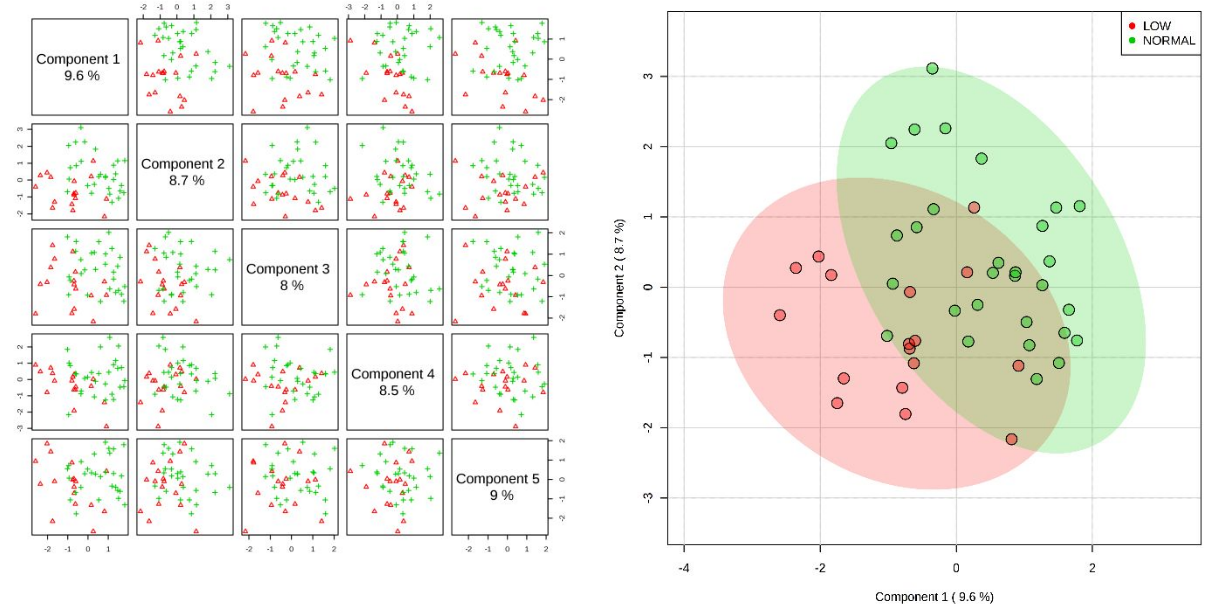

Supplement: Supplementary file 1 — Appendix S1 : Supporting information. [file JBM4-4-e10372-s001.pdf]
